# Supplementary material for: Stress Responses in Horses Housed in Different Stable Designs during Summer in a Tropical Savanna Climate
Source: Animals (Basel). 2024 Aug 4;14(15):2263. doi: 10.3390/ani14152263 (PMC11311062; doi:10.3390/ani14152263)
Supplement: Supplementary file 1 [file animals-14-02263-s001.zip › animals-3123744-supplementary.pdf]

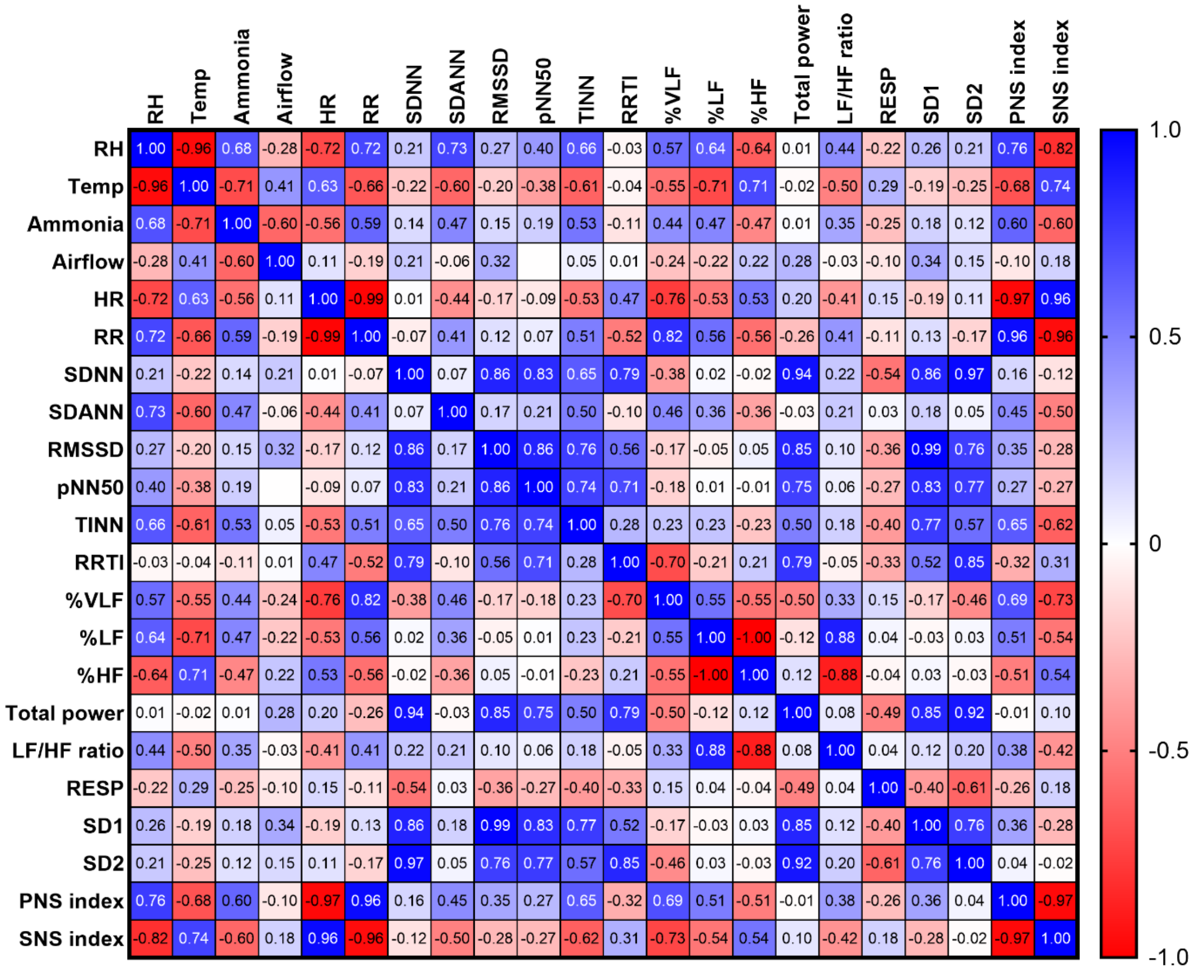

	RH	Temp	Ammonia	Airflow	HR	RR	SDNN	SDANN	RMSSD	pNN50	TINN	RRTI	%VLF	%LF	%HF	Total power	LF/HF ratio	RESP	SD1	SD2	PNS index	SNS index
RH	1.00	-0.96	0.68	-0.28	-0.72	0.72	0.21	0.73	0.27	0.40	0.66	-0.03	0.57	0.64	-0.64	0.01	0.44	-0.22	0.26	0.21	0.76	-0.82
Temp	-0.96	1.00	-0.71	0.41	0.63	-0.66	-0.22	-0.60	-0.20	-0.38	-0.61	-0.04	-0.55	-0.71	0.71	-0.02	-0.50	0.29	-0.19	-0.25	-0.68	0.74
Ammonia	0.68	-0.71	1.00	-0.60	-0.56	0.59	0.14	0.47	0.15	0.19	0.53	-0.11	0.44	0.47	-0.47	0.01	0.35	-0.25	0.18	0.12	0.60	-0.60
Airflow	-0.28	0.41	-0.60	1.00	0.11	-0.19	0.21	-0.06	0.32		0.05	0.01	-0.24	-0.22	0.22	0.28	-0.03	-0.10	0.34	0.15	-0.10	0.18
HR	-0.72	0.63	-0.56	0.11	1.00	-0.99	0.01	-0.44	-0.17	-0.09	-0.53	0.47	-0.76	-0.53	0.53	0.20	-0.41	0.15	-0.19	0.11	-0.97	0.96
RR	0.72	-0.66	0.59	-0.19	-0.99	1.00	-0.07	0.41	0.12	0.07	0.51	-0.52	0.82	0.56	-0.56	-0.26	0.41	-0.11	0.13	-0.17	0.96	-0.96
SDNN	0.21	-0.22	0.14	0.21	0.01	-0.07	1.00	0.07	0.86	0.83	0.65	0.79	-0.38	0.02	-0.02	0.94	0.22	-0.54	0.86	0.97	0.16	-0.12
SDANN	0.73	-0.60	0.47	-0.06	-0.44	0.41	0.07	1.00	0.17	0.21	0.50	-0.10	0.46	0.36	-0.36	-0.03	0.21	0.03	0.18	0.05	0.45	-0.50
RMSSD	0.27	-0.20	0.15	0.32	-0.17	0.12	0.86	0.17	1.00	0.86	0.76	0.56	-0.17	-0.05	0.05	0.85	0.10	-0.36	0.99	0.76	0.35	-0.28
pNN50	0.40	-0.38	0.19		-0.09	0.07	0.83	0.21	0.86	1.00	0.74	0.71	-0.18	0.01	-0.01	0.75	0.06	-0.27	0.83	0.77	0.27	-0.27
TINN	0.66	-0.61	0.53	0.05	-0.53	0.51	0.65	0.50	0.76	0.74	1.00	0.28	0.23	0.23	-0.23	0.50	0.18	-0.40	0.77	0.57	0.65	-0.62
RRTI	-0.03	-0.04	-0.11	0.01	0.47	-0.52	0.79	-0.10	0.56	0.71	0.28	1.00	-0.70	-0.21	0.21	0.79	-0.05	-0.33	0.52	0.85	-0.32	0.31
%VLF	0.57	-0.55	0.44	-0.24	-0.76	0.82	-0.38	0.46	-0.17	-0.18	0.23	-0.70	1.00	0.55	-0.55	-0.50	0.33	0.15	-0.17	-0.46	0.69	-0.73
%LF	0.64	-0.71	0.47	-0.22	-0.53	0.56	0.02	0.36	-0.05	0.01	0.23	-0.21	0.55	1.00	-1.00	-0.12	0.88	0.04	-0.03	0.03	0.51	-0.54
%HF	-0.64	0.71	-0.47	0.22	0.53	-0.56	-0.02	-0.36	0.05	-0.01	-0.23	0.21	-0.55	-1.00	1.00	0.12	-0.88	-0.04	0.03	-0.03	-0.51	0.54
Total power	0.01	-0.02	0.01	0.28	0.20	-0.26	0.94	-0.03	0.85	0.75	0.50	0.79	-0.50	-0.12	0.12	1.00	0.08	-0.49	0.85	0.92	-0.01	0.10
LF/HF ratio	0.44	-0.50	0.35	-0.03	-0.41	0.41	0.22	0.21	0.10	0.06	0.18	-0.05	0.33	0.88	-0.88	0.08	1.00	0.04	0.12	0.20	0.38	-0.42
RESP	-0.22	0.29	-0.25	-0.10	0.15	-0.11	-0.54	0.03	-0.36	-0.27	-0.40	-0.33	0.15	0.04	-0.04	-0.49	0.04	1.00	-0.40	-0.61	-0.26	0.18
SD1	0.26	-0.19	0.18	0.34	-0.19	0.13	0.86	0.18	0.99	0.83	0.77	0.52	-0.17	-0.03	0.03	0.85	0.12	-0.40	1.00	0.76	0.36	-0.28
SD2	0.21	-0.25	0.12	0.15	0.11	-0.17	0.97	0.05	0.76	0.77	0.57	0.85	-0.46	0.03	-0.03	0.92	0.20	-0.61	0.76	1.00	0.04	-0.02
PNS index	0.76	-0.68	0.60	-0.10	-0.97	0.96	0.16	0.45	0.35	0.27	0.65	-0.32	0.69	0.51	-0.51	-0.01	0.38	-0.26	0.36	0.04	1.00	-0.97
SNS index	-0.82	0.74	-0.60	0.18	0.96	-0.96	-0.12	-0.50	-0.28	-0.27	-0.62	0.31	-0.73	-0.54	0.54	0.10	-0.42	0.18	-0.28	-0.02	-0.97	1.00

Figure S1. Correlation between horses' HRV and environmental parameters in the stable A. The correlation coefficients were determined as weak ($\pm 0.10 \leq r_s < \pm 0.40$), moderate ($\pm 0.40 \leq r_s < \pm 0.70$), strong ($\pm 0.70 \leq r_s < \pm 0.90$), or very strong ($r_s \geq \pm 0.90$) correlations**.** **RT**: relative humidity, **Temp**: air temperature, **HR**: heart rate, **RR**: beat-to-beat intervals, **SDNN**: standard deviation of RR intervals, **SDANN**: standard deviation of the averages of RR intervals in 5-min segments, **RMSSD**: root mean square of successive differences between RR intervals, **pNN50**: relative number of successive RR interval pairs that differ by more than 50 ms, **TINN**: triangular interpolation of normal-to-normal intervals, **RRTI**: RR triangular index, **VLF**: very-low-frequency band, **LF**: low-frequency band, **HF**: high-frequency band, **RESP**: respiratory rate, **SD1**: standard deviation of the Poincaré plot perpendicular to the line of identity, **SD2**: standard deviation of the Poincaré plot along the line of identity, **PNS**: parasympathetic nervous system, **SNS**: sympathetic nervous system.

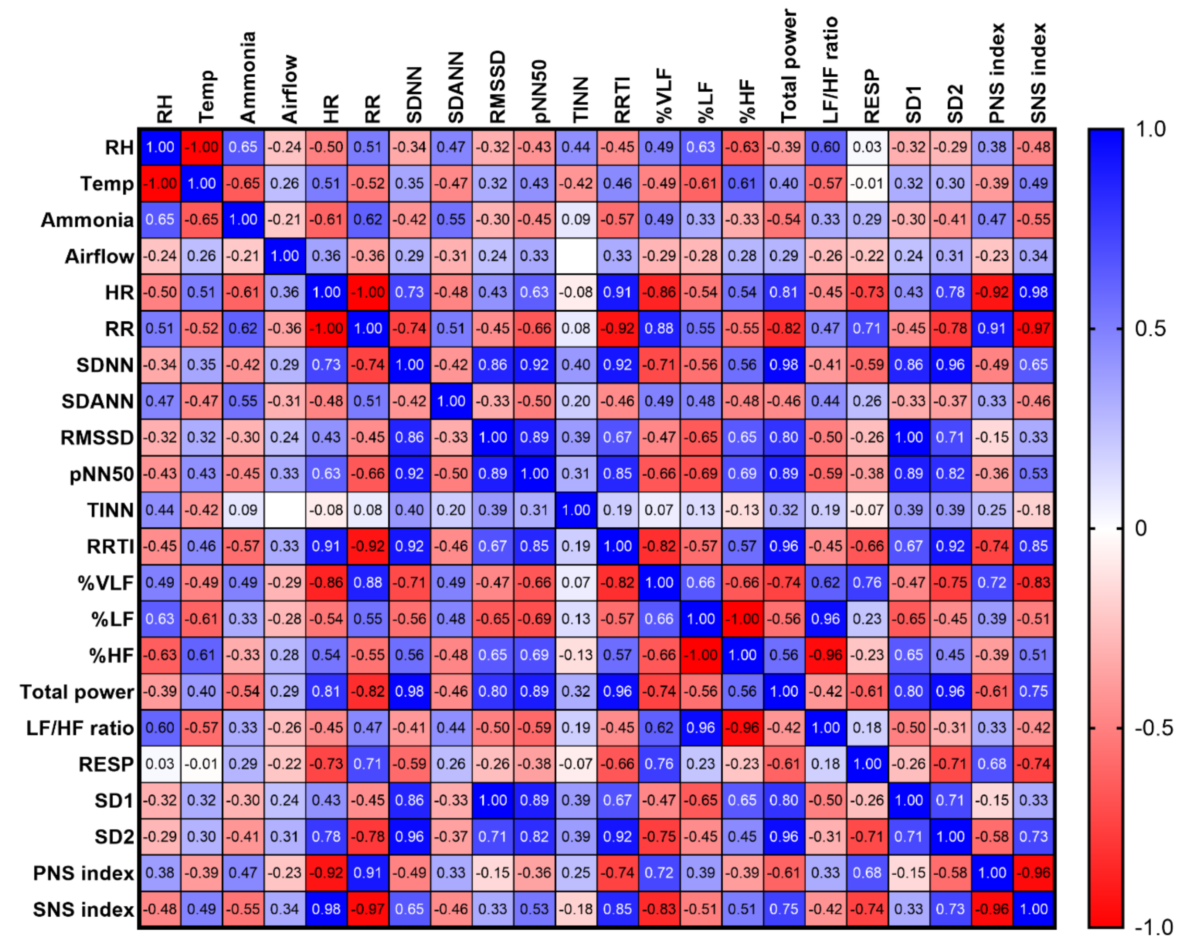

Figure S2. Correlation between horses' HRV and environmental parameters in the stable B. The correlation coefficients were determined as weak (±0.10 ≤ r_s < ±0.40), moderate (±0.40 ≤ r_s < ±0.70), strong (±0.70 ≤ r_s < ±0.90), or very strong (r_s ≥ ±0.90) correlations**.** **RT**: relative humidity, **Temp**: air temperature, **HR**: heart rate, **RR**: beat-to-beat intervals, **SDNN**: standard deviation of RR intervals, **SDANN**: standard deviation of the averages of RR intervals in 5-min segments, **RMSSD**: root mean square of successive differences between RR intervals, **pNN50**: relative number of successive RR interval pairs that differ by more than 50 ms, **TINN**: triangular interpolation of normal-to-normal intervals, **RRTI**: RR triangular index, **VLF**: very-low-frequency band, **LF**: low-frequency band, **HF**: high-frequency band, **RESP**: respiratory rate, **SD1**: standard deviation of the Poincaré plot perpendicular to the line of identity, **SD2**: standard deviation of the Poincaré plot along the line of identity, **PNS**: parasympathetic nervous system, **SNS**: sympathetic nervous system.

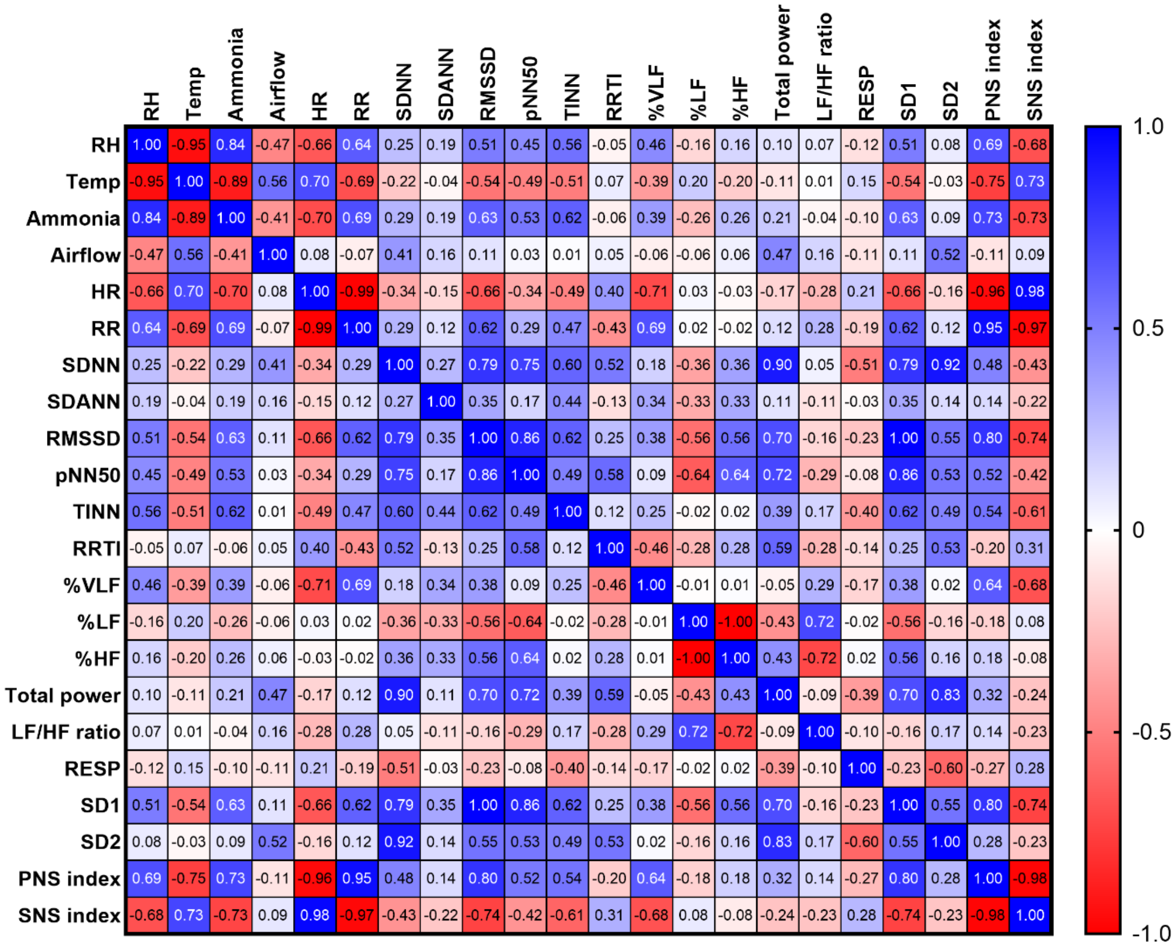

Figure S3. Correlation between horses' HRV and environmental parameters in the stable C. The correlation coefficients were determined as weak ($\pm 0.10 \leq r_s < \pm 0.40$), moderate ($\pm 0.40 \leq r_s < \pm 0.70$), strong ($\pm 0.70 \leq r_s < \pm 0.90$), or very strong ($r_s \geq \pm 0.90$) correlations**.** **RT**: relative humidity, **Temp**: air temperature, **HR**: heart rate, **RR**: beat-to-beat intervals, **SDNN**: standard deviation of RR intervals, **SDANN**: standard deviation of the averages of RR intervals in 5-min segments, **RMSSD**: root mean square of successive differences between RR intervals, **pNN50**: relative number of successive RR interval pairs that differ by more than 50 ms, **TINN**: triangular interpolation of normal-to-normal intervals, **RRTI**: RR triangular index, **VLF**: very-low-frequency band, **LF**: low-frequency band, **HF**: high-frequency band, **RESP**: respiratory rate, **SD1**: standard deviation of the Poincaré plot perpendicular to the line of identity, **SD2**: standard deviation of the Poincaré plot along the line of identity, **PNS**: parasympathetic nervous system, **SNS**: sympathetic nervous system.
